# Gene Co-Expression Analysis Reveals the Transcriptome Changes and Hub Genes of Fructan Metabolism in Garlic under Drought Stress

**DOI:** 10.3390/plants12193357

**Published:** 2023-09-22

**Authors:** Qianyi Zhou, Haihong Sun, Guoli Zhang, Jian Wang, Jie Tian

**Affiliations:** 1 Key Laboratory of Qinghai Tibetan Plateau Biotechnology, Ministry of Education, Academy of Agricultural and Forestry Sciences of Qinghai University, Xining 810016, China; z763335329@163.com (Q.Z.); 1995990031@qhu.edu.cn (H.S.); 15111711276@163.com (G.Z.); 2 Laboratory for Research and Utilization of Germplasm Resources in Qinghai Tibet Plateau, Academy of Agricultural and Forestry Sciences of Qinghai University, Xining 810016, China

**Keywords:** *Allium sativum* L., transcriptome, drought stress, fructan

## Abstract

Drought has become a serious environmental factor that affects the growth and yield of plants. Fructan, as an important storage compound in garlic, plays an important role in drought tolerance. Genomic changes in plants under drought stress clarify the molecular mechanism of plants’ responses to stress. Therefore, we used RNA-seq to determine the transcriptomic changes in garlic under drought stress and identified the key module related to fructan metabolism by weighted gene co-expression network analysis. We conducted a comprehensive analysis of the garlic transcriptome under drought stress over a time course (0, 3, 6, 9, 12, 15 d). Drought significantly induces changes in gene expression. The number of specifically expressed genes were 1430 (3 d), 399 (6 d), 313 (9 d), 351 (12 d), and 1882 (15 d), and only 114 genes responded at each time point. The number of upregulated DEGs was higher than the number of downregulated DEGs. Gene ontology and a Kyoto Encyclopedia of Genes and Genomes analysis showed that garlic was more likely to cause changes in carbohydrate metabolism pathways under drought stress. Fructan content measurements showed that drought stress significantly induced fructan accumulation in garlic. To determine whether there were modules involved in the transcriptional regulation of fructan content in garlic, we further analyzed the genes related to fructan metabolism using WGCNA. They were enriched in two modules, with F-box protein and GADPH as hub genes, which are involved in garlic fructan metabolism in response to drought stress. These results provide important insights for the future research and cultivation of drought-tolerant garlic varieties.

## 1. Introduction

Garlic (*Allium sativum* L.), belonging to the botanical family of Liliaceae, is well-known for its special smell and spicy flavor. As one of the most important vegetables, garlic can not only be used in foods but also can be used for medicinal applications [1]. Garlic is sensitive to moisture stress, especially during bulb development [2]. ‘Ledu purple skin garlic’ is a featured variety in the Qinghai province and is mainly cultivated in Ledu District. Because of the Plateau continental climate, garlic is often subjected to drought stress when it returns to a vegetative phase. This significantly affects the growth and yield of garlic.

Drought is a major abiotic stressor that dramatically affects plant growth and productivity [3]. Due to climate change, drought stress will occur more frequently, and the negative results caused by drought will become even greater [4]. Drought can cause osmotic stress; thus, plants develop protective mechanisms against drought stress, such as accelerating the synthesis of osmoregulatory substances. Sugars, amino acids, and secondary metabolites can help plants maintain cellular homeostasis under drought stress [5,6]. In drought-stressed *Arabidopsis*, soluble sugars make a greater contribution than proline in osmotic regulation [7]. Furthermore, drought stress can also promote the breakdown of storage sugars into soluble sugars (such as fructose and sucrose) [8]. Fructans contribute to a significant part of the dry matter in garlic, accounting for more than 75% of the total dry weight [9]. Many researchers have investigated whether fructans play an important role in plant tolerance to abiotic stresses. Under water stress, fructans increase in *Aloe vera* plants [10]. The expression patterns of related genes have also been explored. *DsSWEET12*, a sugar transporter, improves resistance to osmotic stress in *Arabidopsis* by influencing sugar metabolism [11]. Stress-responsive genes and stress signaling pathways together constitute the plant’s response mechanism to stress [12]. Thus, it is necessary to properly understand the mechanisms of plants under drought stress.

The complexity of drought has been related to the genomic composition of plants [13]. Therefore, molecular approaches have been used to analyze the drought tolerance mechanisms of plants. RNA sequencing (RNA-seq) and weighted gene co-expression network analysis (WGCNA) are widely used to reveal the response mechanisms of plants under drought stress. Genes with similar expression patterns show similar functions and may be involved in or regulate the same signaling pathways. WGCNA is a biological method used to characterize the patterns of gene associations between different samples, identifying highly synergistically varying gene modules based on gene expression patterns and screening hub genes based on connectivity [14]. It has been applied to screen the co-expressed genes responsive to stress in rice [15], maize [16], and sugarcane [17]. By analyzing the hub gene functions of key modules in response to stress, the vital genes of adversity stress can be identified, thus improving crop stress resistance. Dossa et al. [18] detected a hub gene from sesame responsive to stress and demonstrated that the overexpression of this gene in *Arabidopsis* developed tolerance to stress. Yu et al. [19] constructed co-expression networks with low temperature traits in maize. A number of hub genes were screened from four modules with the highest correlations, and these hub genes were closely related to abiotic stress.

An analysis of the transcriptomic changes of hardneck garlic under drought stress during the bolting and bulb stage showed that the upregulated differentially expressed genes (DEGs) were enrichment in the metabolic process, biological process, and carbohydrate metabolism pathways [20]. Wang et al. [21] determined transcriptomic changes in garlic under PEG treatment; the results indicated that the abscisic acid metabolism collaborated with calcium signaling in response to drought stress. At present, the fructan mechanisms of garlic under drought stress mainly focus on changes in the expression patterns of specific genes, but little has been reported to reveal the fructan metabolism response mechanism of garlic to drought from the transcriptome perspective. This study conducted a differential expression analysis of the transcriptome data of garlic leaves treated with drought at different time points. We identified the biological processes at the core of the response. Furthermore, using WGCNA, we associated gene expression modules with fructose content that was constructed of a core gene co-expression network related to the differential expression of fructose metabolism during drought resistance in garlic seedlings. Finally, we proposed a possible novel drought stress response mechanism with unknown genes that could be used to improve drought resistance in garlic and provide new clues and ideas for an in-depth study of the molecular mechanism of garlic fructan metabolism in drought tolerance.

## 2. Results

### 2.1. Transcriptome Sequencing Results and Gene Annotation

We used RNA-seq to enable the comparison of the differential treatment responses under drought. Table S1 shows an overview of the RNA-seq reads for 18 libraries. The average number of clean reads after filtering was higher than 97%. The Q20 (the ratio of the total number of double integers and integer multiples of mass values greater than 20) was higher than 94%, and Q30 was higher than 91%. The percentage of G and C connected in series (GC%) was about 44%.

The length of the transcripts and the clustering sequence were calculated. The results are shown in Table S2. Using the Trinity method with default parameters, these high-quality reads were finally assembled into 444,865. All unigenes were longer than 200 bp, and the N50 of the final assembled transcripts was about 1374–1512 bp. These indicators reflected the high-quality data obtained by sequencing, ensuring reliability in subsequent analyses.

A total of 604,918 transcripts with an N50 of 1374 bp and N90 of 373 bp were generated by Trinity (Table S2). Among them, there were 444,865 unigenes, of which 123,318 had less than 500 bp, 139,092 had 500–1000 bp, 123,842 had 1–2 kb, and 58,613 had >2 kb.

**Figure 1:**
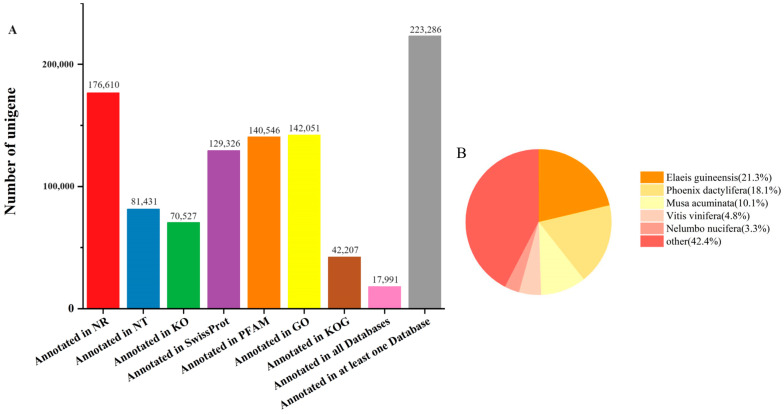
Information on unigenes annotated in different databases (**A**) and distributed in different species (**B**).

**Figure 2:**
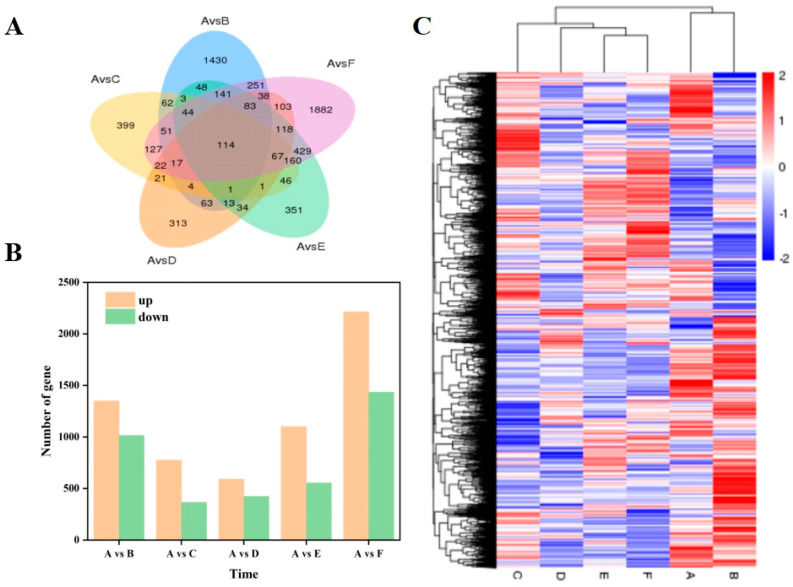
Changes in differentially expressed genes at various time points after drought stress. (**A**) A Venn diagram of differentially expressed genes; (**B**) The number of up- and down-regulated genes in each period; (**C**) A cluster analysis of DEGs in each period (red indicates high expression, and blue indicates low expression; A: 0 days; B: drought stress-3 days; C: drought stress-6 days; D: drought stress-9 days; E: drought stress-12 days; F: drought stress-15 days).

To investigate the genes’ functions, all the assembled unigenes were annotated in the NR, NT, KO, SwissProt, PFAM, GO, and KOG databases (see Section 4). A total of 223,286 unigenes were annotated in at least one database, which accounted for 50.19% of the unigenes (Figure 1A). A total of 17,991 (4.04%) unigenes were annotated in the public databases (Figure 2A). The numbers of unigenes in NR, NT, KO, SwissProt, PFAM, GO, and KOG databases were 176,610 (39.69%), 81,431 (18.30%), 70,527 (15.85%), 129,326 (29.07%), 140,546 (31.59%), 142,051 (31.93%), and 42,207 (9.48%), respectively (Figure 2A). The unigenes matched sequences from the genomes of *Elaeis guineensis*, *Phoenix dactylifera*, *Musa acuminata*, *Vitis vinifera*, *Nelumbo nucifera*, and others (Figure 1B).

### 2.2. The Identification of DEGs under Drought Stress

In this study, DEGs between samples were defined based on the values of fold-change in the expression of assembled transcripts. We defined DEGs with the following criteria: |log2(fold-change) > 1| and corrected *p*-value < 0.05. Throughout drought stress, relative to 0 d (A), specific genes were expressed at different time points: 3 (B), 6 (C), 9 (D), 12 (E), and 15 d (F). There were 1430, 399, 313, 351, and 1882 expressed genes, and only 114 genes responded at each time point (Figure 2A). The number of upregulated DEGs was greater than the number of downregulated DEGs, but the number of downregulated DEGs at 6 and 15 d was greater than the number of upregulated DEGs (Figure 2B), demonstrating that the leaves of garlic seedlings at different stress stages could regulate the response to drought stress by inducing specific genes.

To compare the transcriptomes in differential treatments, we generated a heat map to present the transcript abundance for all DEGs under drought stress (Figure 2C). The results also demonstrated that a range of changes in gene expression occurred when plants were subjected to drought treatment. DEGs at 0 and 3 d clustered together in the early stage of drought stress, while the DEGs of the clustered stage (6 d) and later stage (9, 12, and 15 d) clustered together, indicating that the leaves of garlic seedlings showed significantly different response mechanisms in the early stages of response compared to the middle and late stages.

### 2.3. GO Enrichment Analysis

**Figure 3:**
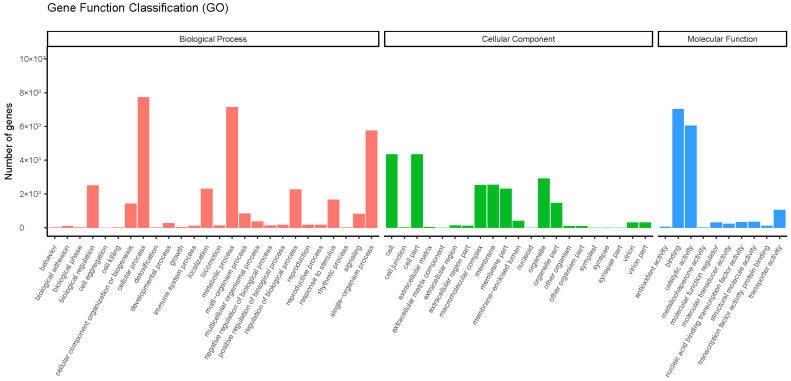
GO classification of DEGs.

To identify the functions of DEGs and the main biological processes involved under drought stress, we performed GO enrichment analysis of DEGs at different stress times, depending on FDR < 0.05. According to GO classification, the differential genes were divided into three groups: molecular function, cellular component, and biological process (Figure 3). In the biological process, 71,500 DEGs were associated with the metabolic process. Drought stress greatly affected the number of DEGs involved in metabolic processes in garlic cells, indicating that drought stress causes changes in various primary and secondary metabolic processes in garlic leaves.

### 2.4. KEGG Pathway Enrichment Analysis

**Figure 4:**
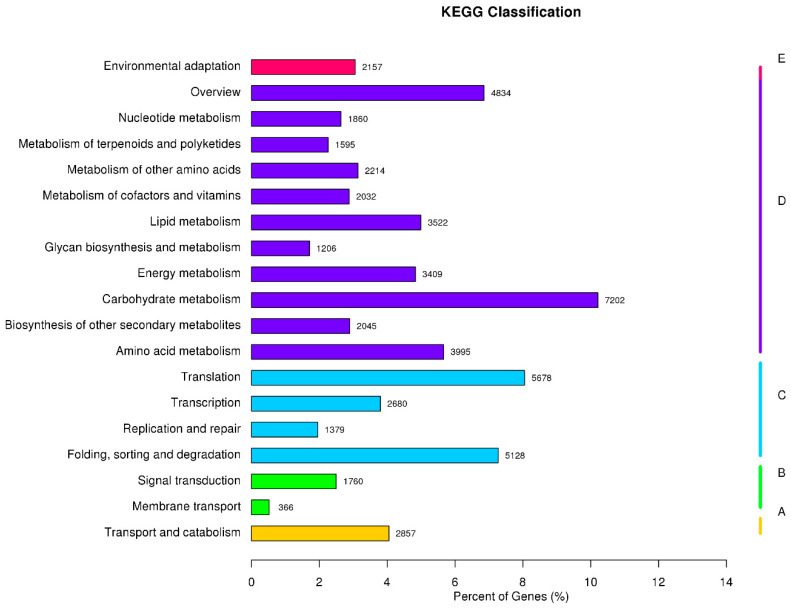
KEGG pathway enrichment analysis of DEGs. A: Cellular Processes, B: Environmental Information Processing, C: Genetic Information Processing, D: Metabolism, E: Organic Systems.

A total of 70,527 unigenes were annotated to the KEGG database (Figure 4), which were then divided into the following five main biochemical metabolic pathways: Cellular Processes, Environmental Information Processing, Genetic Information Processing, Metabolism, and Organic Systems. Among them, metabolic pathways accounted for the largest proportion, mainly including nucleotide metabolism, the metabolism of terpenoids and polyketides, the metabolism of other amino acids, the metabolism of cofactors and vitamins, lipid metabolism, the synthesis and metabolism of polysaccharides, energy metabolism, carbohydrate metabolism, the biosynthesis of secondary metabolism, and amino acid amine metabolism pathways, of which unigenes annotated the largest number of carbohydrate metabolism pathways, indicating that garlic is more likely to cause changes in carbohydrate metabolism pathways under drought stress.

### 2.5. The Variations of Fructan Content in Garlic under Drought Stress

**Figure 5:**
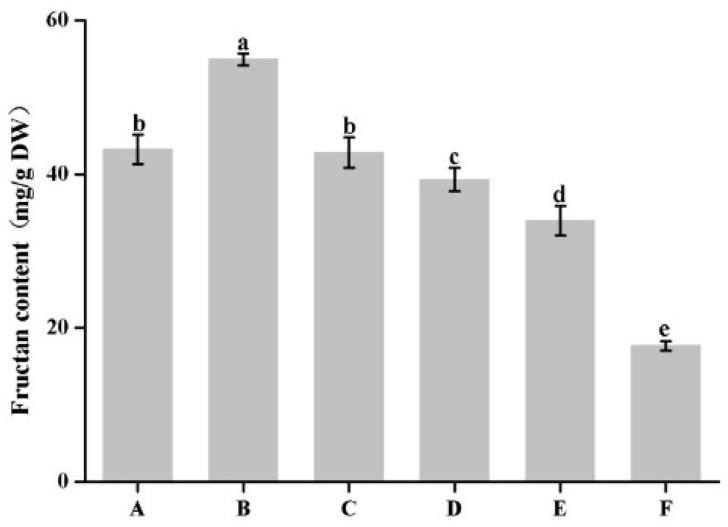
The fructan contents of garlic under drought stress. Different lowercased letters in the same treatment indicate statistical significance (*p* < 0.05). A: 0 d, B: drought stress-3 d, C: drought stress-6 d, D: drought stress-9 d, E: drought stress-12 d, F: drought stress-15 d.

The fructan content of garlic leaves under drought stress were determined (Figure 5). Compared with the control level (A), drought-treated plants showed a significant increase in fructan content at B (3 d) and decrease at D (9 d), E (12 d), and F (15 d), whereas C (6 d) was similar to that in the control level. This showed that garlic responds to oxidative damage from drought stress by mainly inducing fructan synthesis and catabolism.

### 2.6. The Weighted Gene Co-Expression Network Analysis and Functional Annotation of Hub Genes 

**Figure 6:**
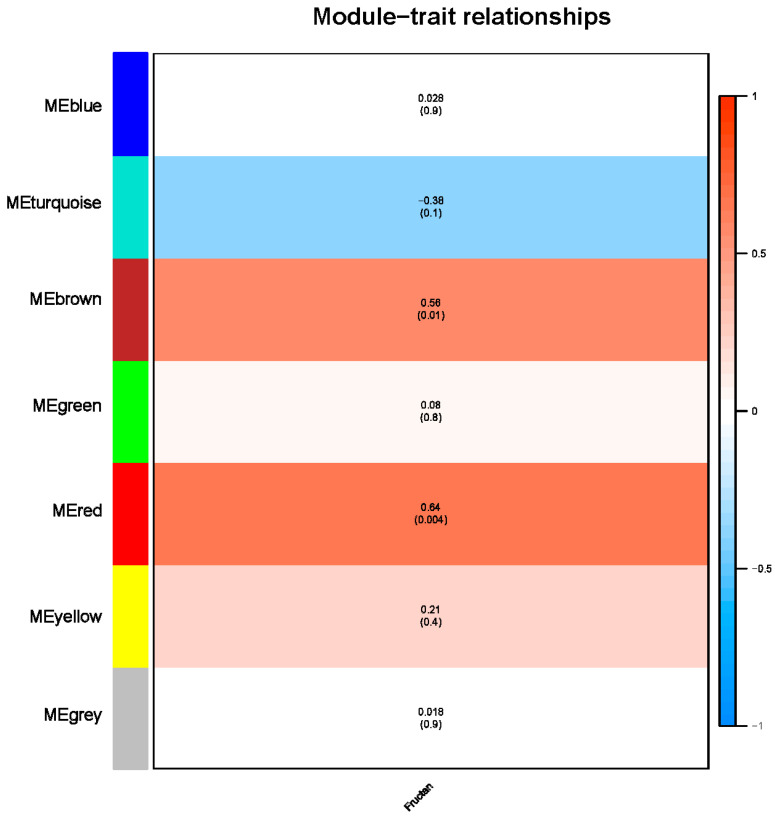
Module–trait relationship with fructan contents. The number represents the correlation coefficient of modules with fructan contents. The number in the brackets indicates the *p*-value.

To determine whether there were modules involved in the transcriptional regulation of fructan content in garlic, all DEGs were used for the construction of the co-expression network (Figure 6). Subsequently, an eigengene-trait correlation analysis was performed for the fructan content. Investigating the relationships between module and fructan content showed that the correlation coefficients varied widely from −0.38 to 0.64. Furthermore, the fructan content was positively correlated with the MEred and MEbrown modules (correlation coefficient > 0.5).

**Figure 7:**
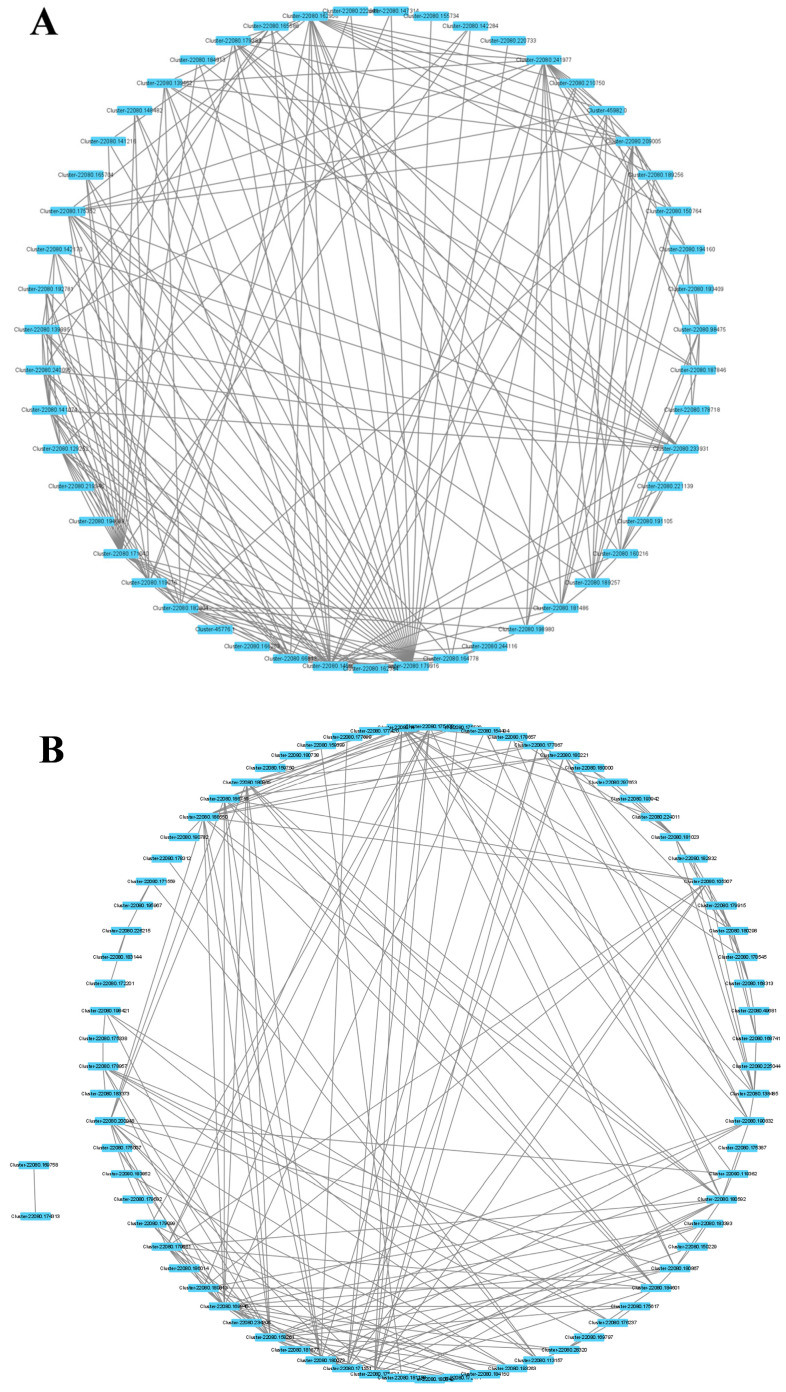
A network analysis of the hub genes in MEred and MEbrown modules that had a high correlation coefficient (0.56–0.64) related to fructan contents. (**A**) MEred module; (**B**) MEbrown module.

The genes related to the fructan contents of garlic were mainly concentrated in the MEred and MEbrown modules. Therefore, the genes with a higher connectivity in the MEred and MEbrown modules were selected to construct the connectivity network, and the hub genes in the network were analyzed (Figure 7). Genes encoding proline-rich protein 4-like, fructokinase-1-like, and proteins of unknown function, including Cluster-22080.233931, Cluster-22080.171040, Cluster-22080.1816132, Cluster-22080.171101, and Cluster-22080.158261 had the highest degree in two modules (Table 1).

## 3. Discussion

Drought is an important environmental factor limiting plant growth and development. Plants have developed protection mechanisms at morphological and biochemical levels to avoid stress damage [22]. With the widespread use of more advanced sequencing technologies, an increasing number of biotechnologies are being applied to reveal the molecular mechanisms of plant responses to drought stress. To reveal the mechanisms of drought resistance in garlic, we investigated molecular changes during different periods of drought stress using RNA-seq. A total of 114 DEGs were identified that corresponded to different stages. The difference between the number of upregulated genes and downregulated genes demonstrated that drought stress induced a positive response for genes in garlic leaves. The results of the clustering analysis of DEGs showed a remarkable difference between the early stages of stress and the late stages. Go and KEGG enrichment analysis demonstrated that drought stress caused changes in metabolic processes, especially in the carbohydrate metabolism pathways of garlic. Carbohydrate metabolism is one of the most important biological processes in plants under abiotic stress. Stress promotes the accumulation of carbohydrates [23]. It has been reported that drought stress increased the total sugar, sucrose, fructose, and glucose content in *Averrhoa carambola* [24]. Zhang et al. [25] found 17 carbohydrate metabolic pathways enriched in the biological metabolism during the response to salt stress in tomatoes, and six of them were significantly enriched. Carbohydrates, which are key to garlic yield formation, are the storage material with the highest dry matter content in the bulbs of garlic. Fructan is one of the most important carbohydrates in garlic, and its accumulation can improve the plant’s resistance to stress. In this study, we compared the fructan content of garlic at different times under drought stress. The results showed that fructan metabolism responded positively to drought stress, and its content peaked at 3 d of drought stress. However, after 6 d of drought treatment, the fructan content showed a decreasing trend. Similarly, the fructan content of wheat under drought stress showed the same trend [26]. When plants are subjected to drought stress, the redistribution of carbohydrates plays an important role in yield [27]. The reduction in fructan content at the late stage of stress may be a result of translocation to storage organs to compensate for the loss of yield due to stress. 

WGCNA provides a new method for elucidating the mechanism of fructan metabolism in response to drought stress. We obtained the key modules related to fructan content under drought stress, and the connectivity of characterized genes in the module allowed key genes in the network to be screened. In this study, two modules that were highly correlated with fructan content were identified, and the genes with the top 10 connectivity in the modules were screened as hub genes. The hub genes of the red module and brown module embodied different biological functions. We identified an F-box protein gene (Cluster-22080.233931) and a methylesterase gene (Cluster-22080.171040) from the red module. An et al. [28] identified and cloned an F-box protein *MdMAX2* from an apple tree. The ectopic expression of *MdMAX2* in *Arabidopsis* exhibited photomorphogenesis phenotypes, and *MdMAX2*-overexpressing apple calli and *Arabidopsis* exhibited increased tolerance to salt and drought stress. From the brown module, we mainly identified glyceraldehyde-3-phosphate dehydrogenase (Cluster-22080.171101) and fructokinase (Cluster-22080.158261). GADPH, a key enzyme involved in glycolysis and gluconeogenesis in plants, and GADPH genes play an important role in carbon metabolism, plant development, and stress tolerance. Wei et al. [29] performed a genome-wide identification of the poplar GAPDH family and screened for a key gene, *PtGAPC1*. By overexpressing *PtGAPC1*, the content of the lipid and carbohydrate metabolites in the plants was significantly changed, indicating that *PtGAPC1* plays an important role in metabolic regulation. Fructokinase is a key enzyme that catalyzes fructose metabolism and plays a role in plant growth and development and stress response [30]. Salt stress contributed to the increase in fructokinase enzyme activities and gene expression to regulate carbohydrate metabolic transduction in sugar beets [31]. Seven *CsFRKs* have been identified in tea plants in response to salt, drought, and cold stress [32]. In addition, some of the unknown functional proteins have not been reported to have relevant functions in any plant, but they have a high connectivity in the co-expression network established in this study. It can be speculated that they may have some functional roles in fructan metabolism pathways under drought stress, and their gene functions will be further investigated by molecular biology and biochemical analysis.

## 4. Materials and Methods

### 4.1. Plant Materials and Stress Treatments

‘Ledu Purple Skin Garlic’ was preserved and supplied by the Qinghai Key Laboratory of Vegetable Genetics and Physiology. Garlic bulbs of uniform size, free of disease and breaking dormancy were selected and sown in potting pots (top aperture× bottom aperture× height size, 16 cm × 13 cm × 16 cm) containing a cultivation substrate (grass charcoal:perlite:vermiculite = 2:1:1) and were cultivated in a plant light incubator. The garlic plants were grown at 25 °C under 14 h of light and at 15 °C under 10 h of darkness, with 12,000 l× light intensity and 70% humidity. When the seedlings reached 10–12 cm, they were grouped for treatment. The control group’s incubation conditions did not change. The pots were watered every 2 days until the water ran out. A consistent pot weight was maintaining by watering and weighing the pots and pans, and a soil relative humidity of 100% was maintained. For drought treatments, water was controlled by stopping watering after thoroughly watering the potting bowl until the water ran out before treatment. Relative humidity in the air was 30%, and relative humidity in the soil was 45–55%. Samples were harvested at 0, 3, 6, 9, 12, and 15 d of each treatment. Each sample consisted of leaves from five plants grown under the same conditions. All leaf samples were immediately frozen in liquid nitrogen and stored at −80 °C. Half of the samples were used for transcriptome analysis.

### 4.2. RNA Isolation and Library Preparation for Transcriptomics Analysis

Samples collected at 0, 3, 6, 9, 12, and 15 d for each treatment were selected for cDNA library construction and transcriptome analyses (completed by Novogene Company, Beijing, China). Three biological replicates per treatment were considered. The mRNAs were purified from the total RNA samples using the OligoTex mRNA mini kit (Qiagen, Beijing, China). The mRNA was then fragmented into small pieces using a fragmentation buffer. Using these short fragments as templates, the first cDNA strand was synthesized using random hexamers, and the second cDNA strand was synthesized using buffers, dNTPs, DNA polymerase I, and RNase H. The cDNA fragments were purified using the AMPure XP beads and resolved with EB buffer for end reparation and poly(A) addition. The short fragments were then connected with sequencing adapters, and the products were subsequently purified and amplified via PCR to create the final cDNA libraries. After passing the library inspection, different libraries were pooled according to effective concentration and the target downstream data volume and then subjected to Illumina HiSeq sequencing. 

### 4.3. The Measurement of Fructan Content in Garlic

Fructan concentrations were measured using HPLC (Waters, Alliance HPLC System, Tokyo, Japan). The samples were dried, and 0.84 g of two equal dry samples of garlic leaves were weighed into 25-mL glass test tubes A and B. Twenty milliliters of distilled water was added to test tube A, boiled for 0.5 h, and cooled to an ambient temperature; the supernatant was centrifuged to 25 mL to form sample A to be tested. In test tube B, 17.4 mL of distilled water and 1 mL of 3 mol/L HCl solution were mixed and boiled for 1 h. Then, 1 mL of 3 mol/L NaOH solution and 0.6 mL of 8.75 mol/L Al_2_(SO_4_)_3_ solution were added, shaken well, and centrifuged for a supernatant of 25 mL to form sample B. The two samples were passed through a 0.2 μm microporous filter membrane. Determination was performed on a high performance liquid chromatograph (Shimadzu RID-10A, Tokyo, Japan) using a Shodex SUGAR KS-801 series KS-802 column with a differential detector under the following conditions: ultrapure water as the mobile phase, a flow rate of 1 mL/min, a column temperature of 80 °C, and a sample volume of 5 μL. Quantitative analysis was performed using the standards of each sugar component and the external standard method, and the contents of each sugar component were expressed in mg/g DW. 

### 4.4. Sequencing, Assembly, and Functional Annotation

To acquire comprehensive gene function information, the sequences obtained were annotated in seven major databases (Nr, Nt, Pfam, KOG/COG, Swiss-prot, KEGG, and GO). Transcriptome data were screened for differentially expressed genes at different stages using twice the difference value and significant expression difference *p*-value. The GO database was used to annotate biological functions that were significantly related to DEGs. The KEGG database allowed for systematic analysis of the metabolic pathways of gene products in cells and the functions of these gene products. Therefore, based on the GO and KEGG databases, the functions and pathways of the genes involved in this study were better screened. 

### 4.5. Weighted Correlation Network Analysis

WGCNA was used to correlate gene expression changes with physiological indicators, thus mining hub genes in the process of changing physiological indicators. Based on the transcriptome data, genes with a FPKM less than 0.5 were filtered out, considering that the low expression genes were not biologically significant, and the remaining genes were used as input values for WGCNA (WGCNA v1.69 package in R). A Pearson correlation matrix and network topology analysis were used to determine the gene correlation and soft thresholding power, respectively. The adjacency was converted to a topological overlap matrix. The analysis parameters for the WGCNA were set as follows: the minimum module size was 30, the soft threshold power for the correlation network was set to 7, and the minimum height of the merge module was 0.25. The networks were visualized using Cytoscape v3.8.0. CytoHubba’s MCC algorithm was used to screen for hub genes.

## 5. Conclusions

In conclusion, this research used RNA-seq and WGCNA to analyze the regulatory mechanisms of fructan metabolism under drought stress. GO and KEGG analyses highlighted primary and secondary metabolic processes and carbohydrate metabolism pathways as key biological processes and metabolic pathways involved in drought response in garlic. The results of the fructan content measurement showed that drought stress significantly induced fructan accumulation in garlic. Furthermore, the hub genes related to fructan metabolism are potential targets for engineering garlic with improved drought tolerance. There are more unknown genes in the red module, which affects the screening and identification of key genes for fruit polymerization metabolism under drought stress. Additionally, this result provides a theoretical basis for further investigation of unknown proteins in garlic with reference to drought and other stressors.

## Figures and Tables

**Table 1:** A functional annotation of hub genes in the modules.

Module	Gene ID	Gene Function	PFAM Description
Mered	Cluster-22080.179916	--	PF06954: Resistin
	Cluster-22080.182804	--	--
	Cluster-22080.145924	--	--
	Cluster-22080.66813	--	--
	Cluster-22080.129252	--	--
	Cluster-22080.162958	--	--
	Cluster-22080.119076	--	--
	Cluster-22080.233931	F-box protein PP2-A13-like	
	Cluster-22080.171040	methylesterase 1-like	PF06821: Serine hydrolase
	Cluster-22080.209005	--	--
Mebrown	Cluster-22080.181613	ribulose bisphosphate carboxylase	--
	Cluster-22080.169940	uncharacterized protein ycf39	--
	Cluster-22080.171101	glyceraldehyde-3-phosphate dehydrogenase A	PF02800: Glyceraldehyde 3-phosphate dehydrogenase, C-terminal domain
	Cluster-22080.158261	fructokinase-1-like	PF08013: Tagatose 6 phosphate kinase
	Cluster-22080.180073	traB domain-containing protein	--
	Cluster-22080.188550	carbonic anhydrase	PF00484: Carbonic anhydrase
	Cluster-22080.138485	amino acid permease 6	PF03222: Tryptophan
	Cluster-22080.180592	thioredoxin M-type protein	PF08534: TSA family
	Cluster-22080.175124	glutamine synthetase leaf isozyme	PF03951: Glutamine synthetase
	Cluster-22080.180867	carbonic anhydrase 2 isoform X1	--

## Data Availability

The transcriptome data used in this study are still unpublished.

## Supplementary Materials

The following supporting information can be downloaded at https://www.mdpi.com/article/10.3390/plants12193357/s1, Table S1: Summary of sequencing data quality. Table S2: List of splicing length distribution.
