# Supplementary material for: Gene Co-Expression Analysis Reveals the Transcriptome Changes and Hub Genes of Fructan Metabolism in Garlic under Drought Stress
Source: Plants (Basel). 2023 Sep 22;12(19):3357. doi: 10.3390/plants12193357 (PMC10574564; doi:10.3390/plants12193357)
Supplement: Supplementary file 1 [file plants-12-03357-s001.zip › plants-2528227-supplementary.pdf]

**Table S1** Summary of sequencing data quality

| Sample | Raw Reads | Clean reads | Clean bases | Error(%) | Q20(%) | Q30(%) | GC(%) |
|---|---|---|---|---|---|---|---|
| A_1 | 48882404 | 48300674 | 7.25G | 0.03 | 97.18 | 92.18 | 44.92 |
| A_2 | 43846336 | 42986032 | 6.45G | 0.03 | 95.24 | 93.14 | 44.41 |
| A_3 | 57210364 | 55979060 | 8.4G | 0.03 | 95.47 | 93.45 | 44.76 |
| B_1 | 54257518 | 53353704 | 8G | 0.03 | 94.93 | 92.76 | 44.38 |
| B_2 | 48333432 | 47773134 | 7.17G | 0.03 | 97.08 | 92.01 | 44.48 |
| B_3 | 70107204 | 68709184 | 10.31G | 0.03 | 96.31 | 94.35 | 45.23 |
| C_1 | 45429078 | 44870166 | 6.73G | 0.03 | 97.14 | 92.13 | 45.16 |
| C_2 | 51137978 | 50283984 | 7.54G | 0.03 | 97.18 | 92.23 | 44.38 |
| C_3 | 59574510 | 58446284 | 8.77G | 0.03 | 96.33 | 94.36 | 44.31 |
| D_1 | 46570886 | 45692200 | 6.85G | 0.03 | 95.38 | 93.34 | 45.24 |
| D_2 | 46536406 | 45590504 | 6.84G | 0.03 | 97.15 | 92.08 | 44.65 |
| D_3 | 54457980 | 53217126 | 7.98G | 0.03 | 95.25 | 93.18 | 44.51 |
| E_1 | 52938882 | 51914108 | 7.79G | 0.03 | 95.41 | 93.37 | 44.34 |
| E_2 | 75908614 | 74100432 | 11.12G | 0.03 | 94.89 | 92.73 | 46.52 |
| E_3 | 44781362 | 44144740 | 6.62G | 0.03 | 97.11 | 92.01 | 44.16 |
| F_1 | 55636566 | 54614738 | 8.19G | 0.03 | 96.61 | 91.04 | 44.71 |
| F_2 | 54514410 | 53578788 | 8.04G | 0.03 | 97.58 | 93.13 | 45.46 |
| F_3 | 61489712 | 60443538 | 9.07G | 0.03 | 97.18 | 92.12 | 45.56 |

**Table S2** List of splicing length distribution

| Nucleotides Length(bp) | Transcripts | Unigenes |
|---|---|---|
| 200-500 | 270,563 | 123,318 |
| 500-1000 | 150,345 | 139,092 |
| 1000-2000 | 125,279 | 123,842 |
| >2000 | 58,731 | 58,613 |
| Total | 604,918 | 444,865 |
| Min Length | 201 | 201 |
| Mean Length | 890 | 1,094 |
| Median Length | 574 | 818 |
| Max Length | 14,246 | 14.246 |
| N50 | 1,374 | 1,512 |
| N90 | 373 | 529 |
